# Cardiac Electrophysiologic Effects of Different Local Anesthetics in Langendorff-Perfused Rabbit Hearts

**DOI:** 10.1007/s12012-026-10122-x

**Published:** 2026-05-18

**Authors:** Julian Wolfes, Amy Katthöfer, Felix Wegner, Benjamin Rath, Florian Doldi, Sati Güler-Eren, Lars Eckardt, Gerrit Frommeyer, Christian Ellermann

**Affiliations:** Department of Cardiology II (Electrophysiology), University Hospital Münster, Albert-Schweitzer-Campus 1, 48149 Münster, Germany

## Abstract

Local anesthetics are widely used in medical care. However, their sodium channel blocking properties not only explain their analgesic potency but also their possible cardiotoxic effects. Due to the different pharmacodynamics and pharmacokinetics of different local anesthetics as well as the divergent cellular electrophysiological effects, we aimed to investigate and compare the electrophysiological effects of different local anesthetics in an established Langendorff model of the isolated rabbit heart. 50 hearts of New Zealand White rabbits were retrogradely perfused employing a Langendorff-setup. Eight catheters were placed endo- and epicardially, thereby recording monophasic action potentials. Hearts were paced at seven different cycle lengths (300–900 ms), thus obtaining cycle-length dependent action potential duration at 90% of repolarization (APD_90_), QT intervals and dispersion of repolarization. In addition, burst pacing was utilized to assess ventricular vulnerability. Thereafter, bradycardic AV-blocked hearts were perfused with a hypokalemic solution to enhance the occurrence of triggered activity. After generating baseline data, the hearts were assigned to four groups: In group 1, hearts were treated with 25 µM, and 50 µM lidocaine. Group 2 was perfused with 25 µM, and 50 µM mepivacaine. Group 3 was perfused with 0.5 µM, and 1 µM bupivacaine. Group 4 was perfused with 5 µM, and 10 µM ropivacaine. As expected, perfusion with all local anesthetics led to a significant prolongation of the effective refractory period as a result of sodium channel blockade. Perfusion with bupivacaine and ropivacaine resulted in pronounced cardiotoxic effects, characterized by electromechanical uncoupling or loss-of-capture phenomena, in the majority of hearts. Perfusion with mepivacaine and ropivacaine resulted in the comparatively most pronounced prolongation of the effective refractory period. Accompanied by a pronounced dispersion of repolarization, the highest incidence of ventricular tachycardia and ventricular fibrillation episodes was observed during perfusion with bupivacaine. Perfusion with the local anesthetics showed pronounced electrophysiological effects, which differed between the various local anesthetics. Bupivacaine showed the most pronounced cardiotoxic effects even at low doses.

**Graphical Abstract:**

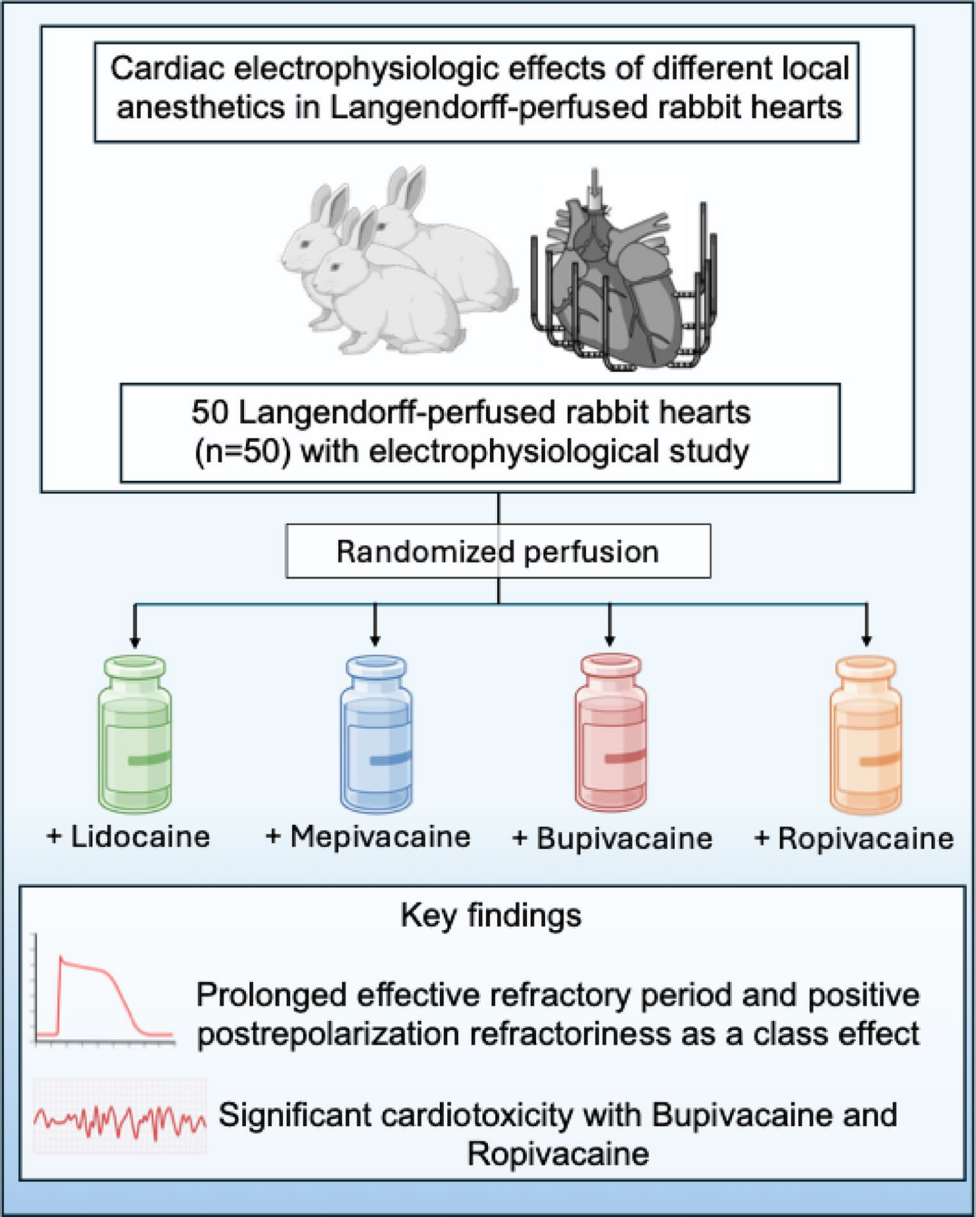

## Introduction

Local anesthetics are widely employed in clinical practice. Lidocaine and bupivacaine are listed among the World Health Organization (WHO) essential medicines [1]. Besides their use as local anesthetics, lidocaine is also employed as a drug therapy for ventricular arrhythmias [2, 3]. Local anesthetics can cause various side effects, including significant cardiotoxicity, during intoxication, such as in cases of accidental intravascular injections. These may include ECG changes [4], ventricular arrhythmias [5], and electromechanical decoupling [6], all of which can often be fatal. Such outcomes can often be prevented using lipid infusions [7, 8] or temporary mechanical circulatory support [9].

Generally, local anesthetics are classified based on their potency, lipid solubility, and duration of action [10]. Less lipophilic and therefore shorter-acting local anesthetics are generally considered safer, particularly with regard to systemic and cardiac toxicity. However, for prolonged regional anesthesia procedures, long-acting and more lipophilic agents such as bupivacaine or ropivacaine are often required. Interestingly, the cardiotoxicity of different local anesthetics varies. Bupivacaine, in particular, is known for its significant cardiotoxicity, which led to the development of its derivative ropivacaine [7]. In contrast, case reports of cardiotoxicity exist for shorter-acting local anesthetics like lidocaine [11] and mepivacaine [9], but they are generally regarded as safer concerning cardiotoxicity, and lidocaine is also employed as an antiarrhythmic agent [3].

Despite this clinical relevance, the comparative cardiac electrophysiological effects of different local anesthetics at a mechanistic level remain incompletely understood. While the cardiotoxic potential of individual agents, particularly bupivacaine, has been documented in clinical case reports and experimental models [4–6], systematic head-to-head comparisons of multiple local anesthetics under standardized, controlled conditions are scarce. In particular, it remains unclear to what extent agents of varying lipophilicity and pharmacokinetics differ in their effects on key electrophysiological parameters such as action potential duration (APD), effective refractory period (ERP), spatial dispersion of repolarization, and post-repolarization refractoriness (PRR) — parameters that are closely linked to the risk of life-threatening ventricular arrhythmias [12, 13]. Furthermore, the relationship between these electrophysiological changes and the propensity for loss-of-capture phenomena, which represent a distinct form of cardiotoxicity, has not been systematically characterized across drug classes.

The Langendorff isolated perfused heart model offers an established and reproducible platform to assess intrinsic cardiac electrophysiology independently of autonomic and hemodynamic confounders, enabling direct mechanistic comparison between agents. Using this model, the present study aimed to provide a comprehensive, side-by-side characterization of the cardiac electrophysiological safety profiles of four clinically relevant local anesthetics at concentrations within the range of peak plasma levels observed in clinical practice.

For this reason, we aimed to investigate and compare the electrophysiological effects of various local anesthetics in an established model of the isolated rabbit heart and compare their electrophysiological safety profiles.

## Methods

The experimental protocol was approved by the local animal welfare office and the state authority (State Office for Nature, Environment, and Consumer Protection North Rhine-Westphalia, File Number: 81-02.05.50.21.004).

Animals were housed in groups of up to six females under standard conditions in an accredited animal facility. All animals used in this study were female New Zealand White rabbits (Charles River) aged at least 3 months and weighing at least 2.5 kg. Prior to euthanasia, all animals received 5,000 IU heparin via the lateral ear vein to prevent clot formation during retrograde perfusion. Euthanasia was performed by intravenous administration of 1.000 mg thiopental (Inresa Arzneimittel GmbH) via the lateral ear vein, followed by exsanguination.

In summary, 50 rabbit hearts were rapidly excised and retrogradely perfused using a Langendorff setup (Hugo Sachs). Prior to AV nodal ablation, all hearts demonstrated sinus rhythm. Following mechanical AV nodal ablation, hearts were allowed to stabilize for 5 min before further experimental procedures were initiated. Hearts that did not exhibit a spontaneous intrinsic escape rhythm following AV nodal ablation were excluded from the study.

For reproducibility and controllability retrograde perfusion was carried out at a controlled temperature (38 °C by water jacketed heated perfusion and water jacketed warming bath) and pressure of 90 mmHg with a modified Krebs-Henseleit buffer (NaCl 118 mM, NaHCO₃ 24.88 mM, d-glucose 5.55 mM, KCl 4.70 mM, Na-pyruvate 2 mM, CaCl_2_ 1.80 mM, KH₂PO₄ 1.18 mM, MgSO_4_ 0.83 mM (all chemicals purchased from Merck). Perfusion with oxygenated perfusate maintained the heart’s vitality and contractility. Seven monophasic action potential catheters were placed epicardially on the heart, and one endocardial catheter was inserted into the left ventricle via the ostia of the pulmonary veins. Additionally, a pseudo 12-lead ECG was recorded from the warming bath surrounding the heart. Mechanical AV nodal ablation was performed. Subsequently, the pacing protocol was initiated:

Stimulation employing an additional epicardial catheter was performed at 7 different cycle lengths ranging from 900 to 300 ms, increasing in 100 ms steps. During stimulation, action potential duration to 90% repolarization (APD_90_) and the QT interval were measured. Subsequently, pacing with a short-coupled extrastimulus was used to determine the effective refractory period (ERP). Additionally, repetitive burst stimulations were applied to assess ventricular vulnerability. This was followed by perfusion with hypokalemic KHB (K(+) 1.5 mM) to evaluate arrhythmia susceptibility in a low-potassium environment. Spatial dispersion of repolarization was calculated as the difference between the maximum and minimum APD_90_ of the eight simultaneously recorded monophasic action potentials. Post-repolarization refractoriness (PRR) was determined by subtracting APD_90_ from ERP.

**Fig. 1:**
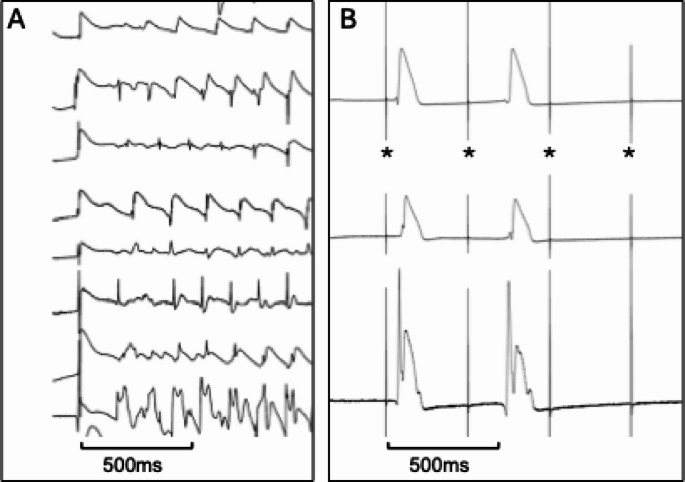
Different types of arrhythmia episodes derived from MAP-recordings observed in rabbit hearts under Langendorff perfusion with various local anesthetics. **A** shows the onset of ventricular tachycardia caused by programmed ventricular stimulation under perfusion with ropivacaine. B depicts a Loss-of-capture (LOC) episode indicated by the absence of ventricular response to ventricular stimulation (*) under perfusion with bupivacaine

Furthermore, episodes of loss of capture (LOC), where stimulation was suddenly lost along with myocardial contraction response, were documented. An example of ventricular tachycardia and LOC under programmed stimulation is shown in Fig. 1.

After establishing baseline electrophysiological parameters, the hearts were divided into four groups. In group 1, 12 hearts (*n* = 12) were perfused with 25 µM lidocaine (medphano Arzneimittel GmbH), then with 50 µM lidocaine. In group 2, 12 hearts (*n* = 12) were perfused with 25 µM and 50 µM mepivacaine (DELTAMEDICA GmbH). In group 3, 13 hearts (*n* = 13) were perfused with 0.5 µM bupivacaine (DELTAMEDICA GmbH), then with 1 µM bupivacaine. In group 4, 13 hearts (*n* = 13) received 5 µM and 10 µM ropivacaine (DELTAMEDICA GmbH). The perfusion was performed for a minimum of 15 min before measurement.

**Fig. 2:**
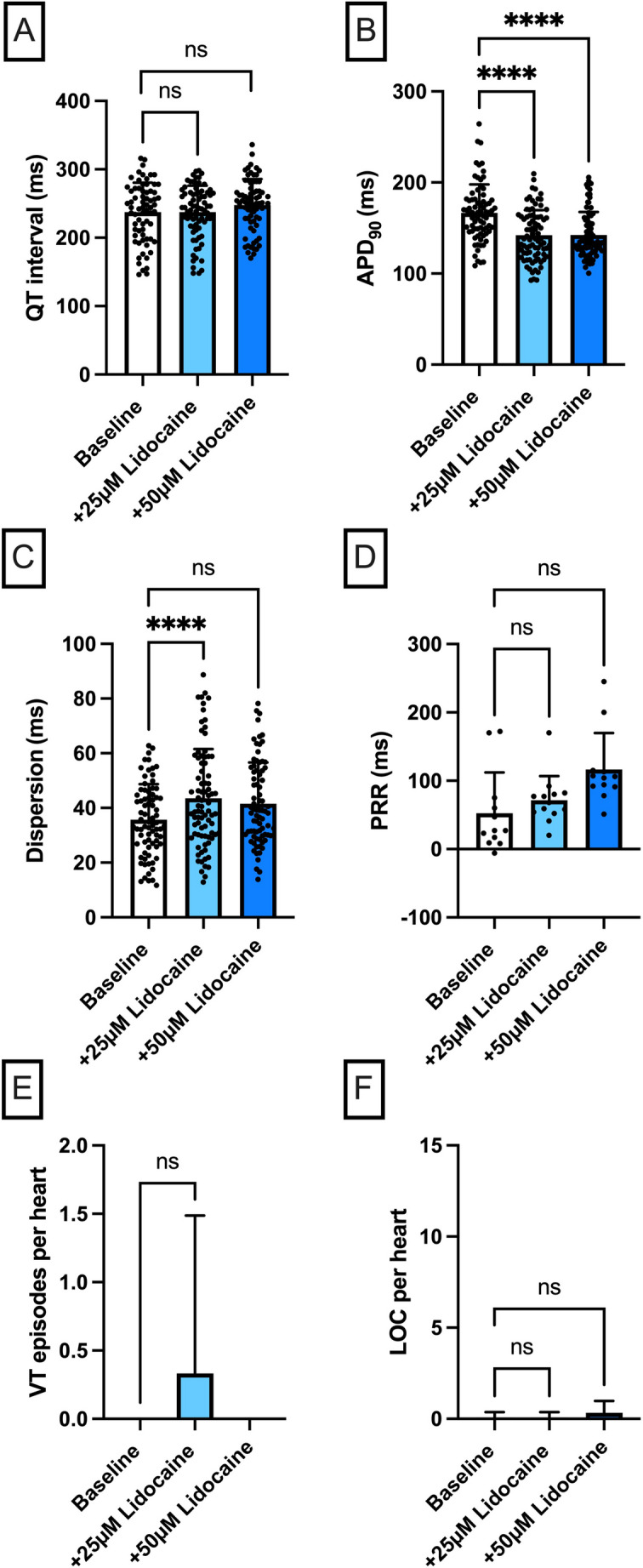
**A** Overall QT interval and **B** APD_90_. Concentration-dependent effect of lidocaine (*n* = 12) on **C** spatial dispersion of repolarization, **D** post-repolarization refractoriness (PRR), **E** number of ventricular tachycardia (VT)/fibrillation (VF) episodes and **F** loss-of-capture (LOC) episodes. The data points in **A–C** represent individual measurements from all seven cycle lengths (900 to 300 ms) for each heart; since each of the n hearts was measured at up to seven cycle lengths, the total number of data points per condition may exceed n. The data point in **D** is derived solely from the measurement at 500 ms cycle length (one value per heart), hence the number of data points equals n (*p < 0.05), *ns* non-significant

**Fig. 3:**
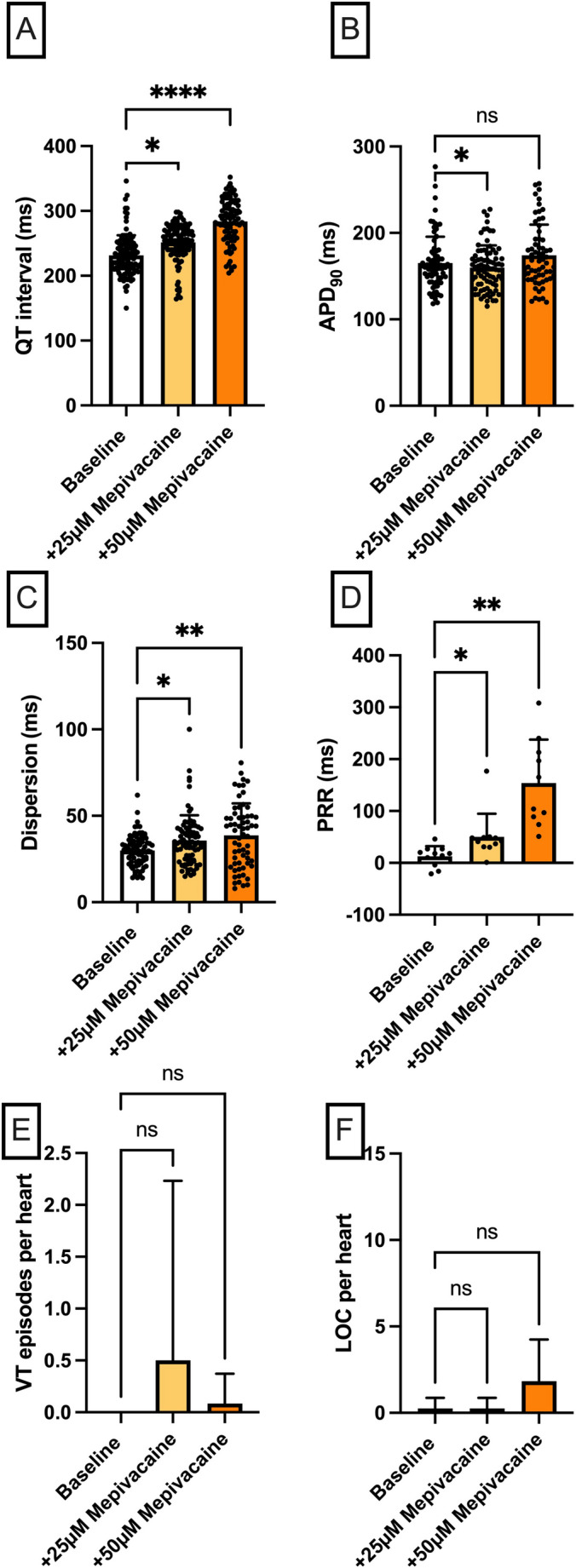
**A** Overall QT interval and **B** APD_90_. Concentration-dependent effect of mepivacaine (*n* = 12) on **C** spatial dispersion of repolarization, **D** post-repolarization refractoriness (PRR), (E) number of ventricular tachycardia (VT)/fibrillation (VF) episodes and **F** loss-of-capture (LOC) episodes. The data points in **A–C** represent individual measurements from all seven cycle lengths (900 to 300 ms) for each heart; since each of the n hearts was measured at up to seven cycle lengths, the total number of data points per condition may exceed n. The data point in **D** is derived solely from the measurement at 500 ms cycle length (one value per heart), hence the number of data points equals n (*p < 0.05), *ns* non-significant

**Fig. 4:**
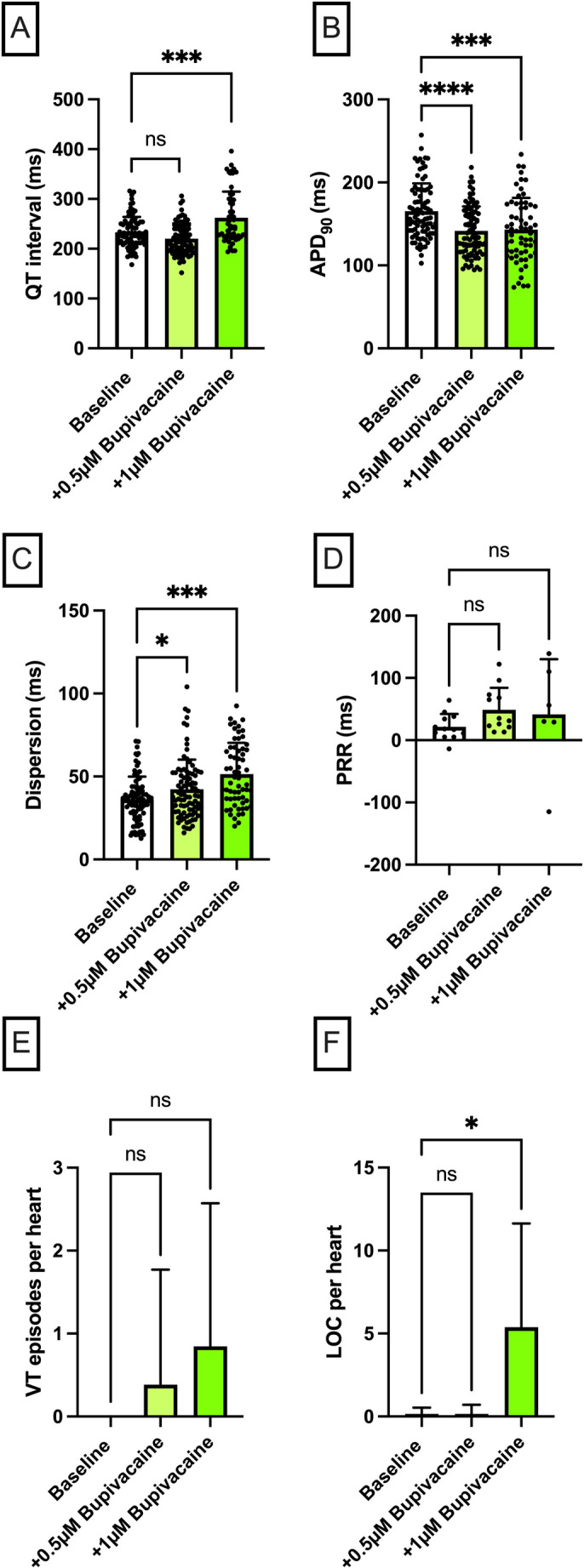
**A** Overall QT interval and **B** APD_90_. Concentration-dependent effect of bupivacaine (*n* = 13) on **C** spatial dispersion of repolarization, **D** post-repolarization refractoriness (PRR), **E** number of ventricular tachycardia (VT)/fibrillation (VF) episodes and **F** loss-of-capture (LOC) episodes. The data points in **A–C** represent individual measurements from all seven cycle lengths (900 to 300 ms) for each heart; since each of the n hearts was measured at up to seven cycle lengths, the total number of data points per condition may exceed n. The data point in **D** is derived solely from the measurement at 500 ms cycle length (one value per heart), hence the number of data points equals n (*p < 0.05), *ns* non-significant

**Fig. 5:**
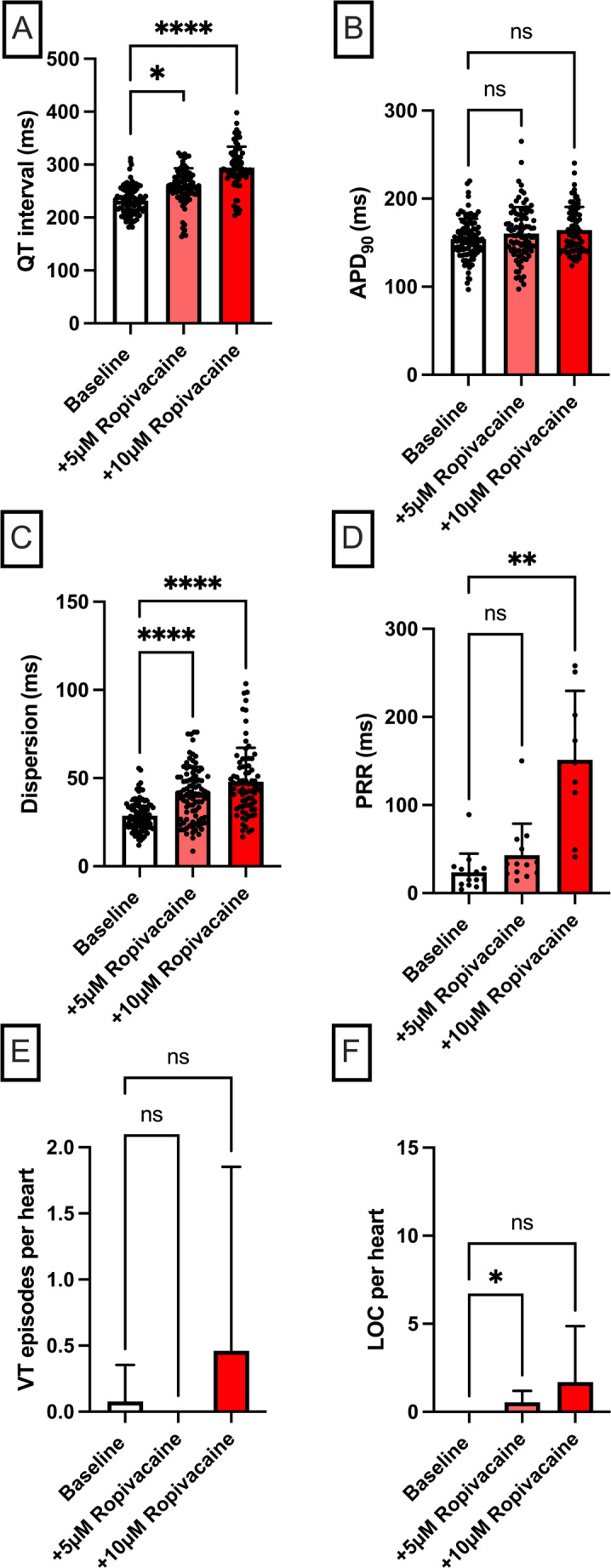
**A** Overall QT interval and **B** APD_90_. Concentration-dependent effect of ropivacaine (*n* = 13) on **C** spatial dispersion of repolarization, **D** post-repolarization refractoriness (PRR), **E** number of ventricular tachycardia (VT)/fibrillation (VF) episodes and **F** loss-of-capture (LOC) episodes. The data points in **A–C** represent individual measurements from all seven cycle lengths (900 to 300 ms) for each heart; since each of the n hearts was measured at up to seven cycle lengths, the total number of data points per condition may exceed n. The data point in **D** is derived solely from the measurement at 500 ms cycle length (one value per heart), hence the number of data points equals n (*p < 0.05), *ns* non-significant

Electrograms and action potentials were recorded on a multi-channel recorder and digitized at 1 kHz with 12-bit resolution. Variables are reported as mean ± standard deviation. Statistical analysis and graphical visualizations were performed using GraphPad Prism version 10. Drug effects on APD_90_, QT interval, spatial dispersion of repolarization, and post-repolarization refractoriness were analyzed using a mixed effects model. P values less than 0.05 were considered statistically significant. Due to the partially missing measured values at different cycle lengths, a variance analysis in the form of an ANOVA (analysis of variance) with repeated measures was not suitable for the evaluation [14]. The exclusion of further measured values caused by the ANOVA would further reduce the data set and thus also significantly influence the statistical evaluation. Instead, the “mixed model” from the program GraphPad Prism (Version 10) was used as an approximation. This model uses a composite symmetric covariance matrix, taking into account “Restricted Maximum Likelihood”. In a data set without missing values, this model behaves very similarly to an ANOVA with measurement reproduction. However, in the case of missing values, it prevents the loss of the entire measurement series of the variable in question. The Geisser–Greenhouse correction was used in the statistical testing. The Tukey method was used to reduce the accumulation of alpha errors [15]. To account for the non-independence of repeated measurements obtained from the same heart at different cycle lengths, drug concentration and cycle length were included as fixed effects in the mixed effects model, with an interaction term to capture cycle-length-dependent drug effects. Individual heart identity was modelled as a random effect (random intercept per subject), thereby explicitly accounting for within-subject correlation across cycle lengths and drug concentrations. No additional covariates were included, as all hearts underwent an identical experimental protocol under standardized conditions. To further guard against pseudoreplication, a confirmatory two-stage analysis was performed: individual measurements were first averaged across all cycle lengths for each heart, yielding a single mean value per animal per condition. A repeated measures analysis of these per-heart means confirmed the results obtained with the mixed effects model, supporting the validity of the primary statistical approach. For post-repolarization refractoriness (panel D in Figs. 2, 3, 4 and 5), measurements were obtained at a single cycle length (500 ms) only, and therefore no averaging step was required.

## Results

### Lidocaine

Perfusion with lidocaine significantly shortened APD_90_ [baseline: 167 ± 31 ms; + 25 µM lidocaine: 142 ± 28 ms (*p* < 0.05); + 50 µM lidocaine: 142 ± 25 ms (*p* < 0.05)], but did not affect the QT interval [baseline: 238 ± 43 ms; + 25 µM lidocaine: 237 ± 39 ms (p = ns); + 50 µM lidocaine: 248 ± 38 ms (p = ns)] (Fig. 2). This was accompanied by increased spatial dispersion, especially between the baseline and 25 µM lidocaine conditions. The ERP [baseline: 184 ± 69 ms; + 25 µM lidocaine: 185 ± 56 ms (p = ns); + 50 µM lidocaine: 225 ± 81 ms (p = ns)] and PRR was slightly but not significantly prolonged. The incidence of spontaneous ventricular arrhythmias (baseline: 0 episodes in 12 hearts; + 25 µM lidocaine: 4 episodes in 12 hearts; + 50 µM lidocaine: 0 episodes in 12 hearts) and loss-of-capture episodes (baseline: 1 episode in 12 hearts; + 25 µM lidocaine: 1 episode in 12 hearts; + 50 µM lidocaine: 4 episodes in 12 hearts) did not change significantly.

### Mepivacaine

Mepivacaine caused a notable prolongation of the QT interval [baseline: 231 ± 31 ms; + 25 µM mepivacaine: 251 ± 28 ms (*p* < 0.05); + 50 µM mepivacaine: 284 ± 34 ms (*p* < 0.05)] (Fig. 3). The APD_90_ was not affected to the same degree [baseline: 165 ± 30 ms; + 25 µM mepivacaine: 160 ± 26 ms (*p* < 0.05); + 50 µM mepivacaine: 174 ± 36 ms (p = ns)]. The dispersion [baseline: 30 ± 9 ms; + 25 µM mepivacaine: 36 ± 15 ms (*p* < 0.05); + 50 µM mepivacaine: 39 ± 19 ms (*p* < 0.05)], the ERP [baseline: 174 ± 13 ms; + 25 µM mepivacaine: 210 ± 43 ms (*p* < 0.05); + 50 µM mepivacaine: 309 ± 91 ms (*p* < 0.05)] and the PRR [baseline: 13 ± 20 ms; + 25 µM mepivacaine: 50 ± 44 ms (*p* < 0.05); + 50 µM mepivacaine: 154 ± 84 ms (*p* < 0.05)] increased significantly with higher doses of mepivacaine. Mepivacaine had no significant effect on the occurrence of VT or LOC episodes.

### Bupivacaine

Perfusion with bupivacaine caused an increase in the QT interval at higher doses [baseline: 233 ± 31 ms; + 0.5 µM: 220 ± 31 ms (p = ns); + 1 µM: 262 ± 53 ms (*p* < 0.05)], while the APD_90_ was significantly shortened [baseline: 165 ± 33 ms; + 0.5 µM: 142 ± 29 ms (*p* < 0.05); + 1 µM: 143 ± 38 ms (*p* < 0.05)] (Fig. 4). The dispersion of repolarization increased significantly in a concentration-dependent manner [baseline: 36 ± 14 ms; + 0.5 µM: 42 ± 18 ms (*p* < 0.05); + 1 µM: 51 ± 19 ms (*p* < 0.05)]. The ERP [baseline: 189 ± 21 ms; + 0.5 µM: 187 ± 18 ms (p = ns); + 1 µM: 240 ± 62 ms (p = ns)] was altered insignificantly, and PRR was altered significantly [baseline: 21 ± 21 ms; + 0.5 µM: 49 ± 35 ms (p = ns); + 1 µM: 42 ± 89 ms (p = ns)]. Ventricular tachycardia episodes slightly increased but did not reach statistical significance (baseline: 1 in 13 hearts; + 0.5 µM: 5 in 13 hearts; + 1 µM: 11 in 13 hearts). Loss-of-capture episodes significantly increased at higher concentrations [baseline: 1 in 13 hearts; + 0.5 µM: 2 in 13 hearts (p = ns); + 1 µM: 70 in 13 hearts (*p* < 0.05)].

### Ropivacaine

Langendorff perfusion with ropivacaine significantly prolonged the QT interval [baseline: 232 ± 27 ms; + 5 µM: 259 ± 34 ms (*p* < 0.05); + 10 µM: 294 ± 40 ms (*p* < 0.05)], while the prolongation of the APD_90_ was non-significant [baseline: 154 ± 23 ms; + 5 µM: 160 ± 30 ms (p = ns); + 10 µM: 164 ± 26 ms (p = ns)] (Fig. 5). The dispersion of repolarization increased significantly in a dose-dependent manner [baseline: 29 ± 9 ms; + 5 µM: 41 ± 15 ms (*p* < 0.05); + 10 µM: 48 ± 19 ms (*p* < 0.05)]. The ERP was significantly prolonged under higher doses [baseline: 176 ± 26 ms; + 5 µM: 200 ± 35 ms (p = ns); + 10 µM: 301 ± 73 ms (*p* < 0.05)], PRR was notably higher at the higher concentration. Although there was a trend toward more VT episodes, it was not statistically significant. Loss-of-capture episodes increased compared to baseline [baseline: 0 in 13 hearts; + 5 µM: 7 in 13 hearts (*p* < 0.05); + 10 µM: 22 in 13 hearts (not significant)].

## Discussion

### Influence of Local Anesthetics on APD_90_ and QT Interval

**Fig. 6:**
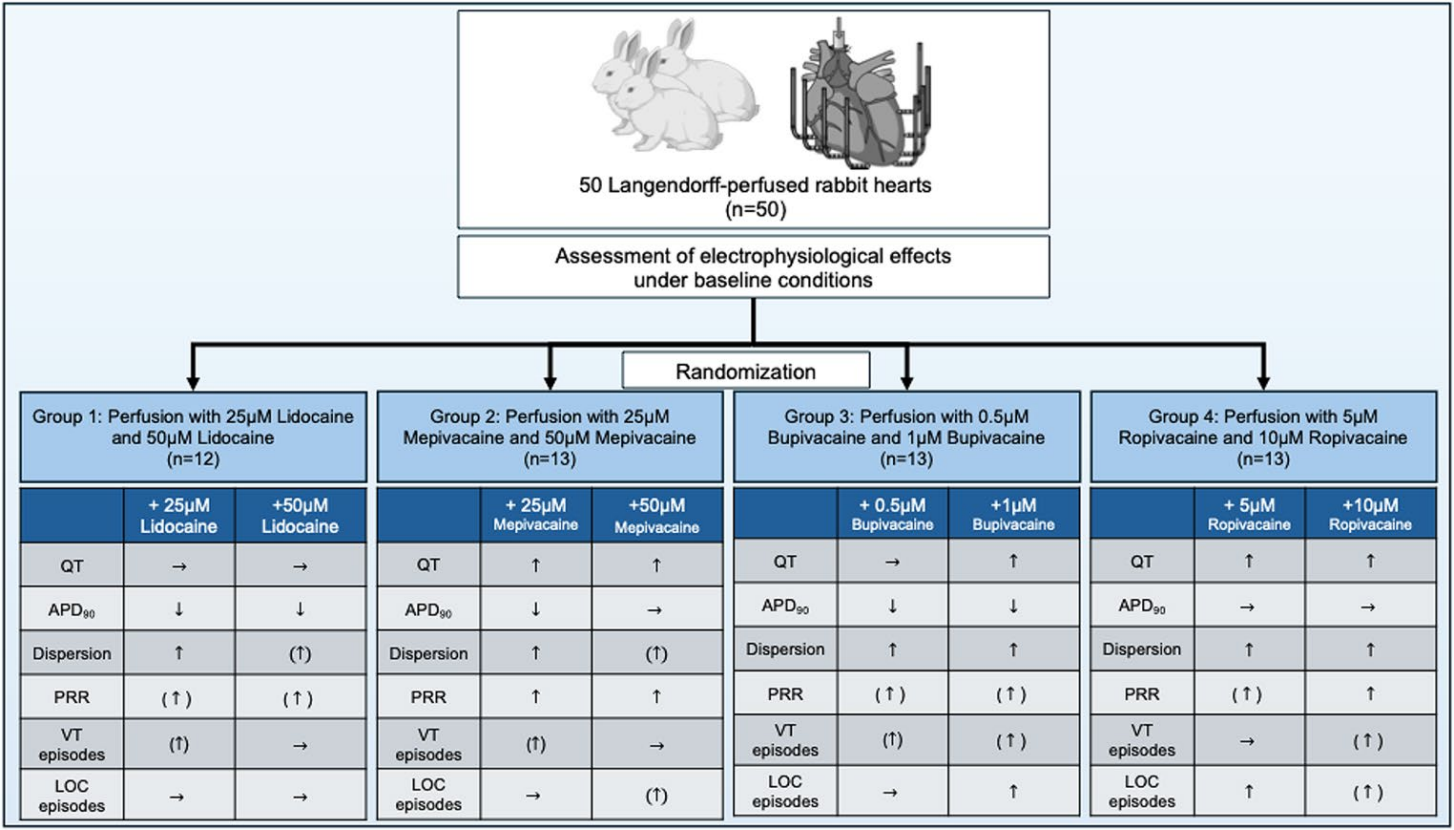
Central illustration on the effects of different local anesthetics on the cardiac electrophysiology in Langendorff-perfused rabbit hearts. Arrows in brackets indicate effects not reaching the threshold for statistical significance. *APD*_90_ action potential duration at 90% of repolarization, *LOC* loss-of-capture, *VT* ventricular tachycardia

Figure 6 summarizes the electrophysiological effects of the different local anesthetics on cardiac electrophysiology. Perfusion with lidocaine, bupivacaine, and low doses of mepivacaine significantly shortened APD_90_. This effect has been previously reported for local anesthetics and has been demonstrated in studies on rabbit Purkinje fibers with lidocaine [16] and mouse cardiomyocytes [17] with comparable concentrations. Where it is attributed to sodium channel inhibition, especially the late sodium influx I_NaL_ [18]. Interestingly, ropivacaine had no effect on myocardial APD_90_ at the concentrations tested. This finding is consistent with published data demonstrating that ropivacaine shortens APD in a concentration-dependent manner, with an EC₅₀ for the reduction of maximal depolarization velocity of approximately 81 µM in isolated canine ventricular cardiomyocytes [19]—considerably above the concentrations employed in the present study (5 and 10 µM). The absence of a significant APD_90_ effect at these lower, clinically relevant concentrations likely reflects insufficient sodium channel blockade to produce measurable APD shortening, which is further supported by the faster dissociation kinetics of ropivacaine from the sodium channel compared to bupivacaine, resulting in less pronounced class Ib antiarrhythmic effects [20].

Conversely, while bupivacaine and ropivacaine inhibit cardiac hERG channels at micromolar concentrations—with greater potency than mepivacaine [21, 22]—lidocaine primarily exerts sodium channel-mediated effects on cardiac electrophysiology. At the concentrations employed in the present study, this potassium channel blockade by ropivacaine may dominate over its weaker sodium channel effects, contributing to QT prolongation.

The apparent paradox of QT interval prolongation, despite shortened APD, can be explained by sodium channel blockade causing decreased ventricular conduction velocity and increased excitation dispersion. These changes can extend the QT interval on surface ECG while APD_90_ remains stable or even shortens [13].

### Effect on Dispersion and Refractory Period

Perfusion with all local anesthetics significantly increased spatial dispersion, especially with bupivacaine and ropivacaine compared to lidocaine and mepivacaine. This rise can be attributed to spatial heterogeneity in the distribution of inhibited sodium channels [4], as shown in large animal studies [23] and clinical observations [24]. The larger effect with bupivacaine and ropivacaine may be due to their longer channel interaction compared to lidocaine and mepivacaine [25]. Additionally, their strong multichannel blocking effect, including hERG channels [21], could contribute to the increased dispersion. When considering the prolongation of the refractory period or the PRR as the ratio of refractoriness to action potential duration, perfusion with these drugs tends to extend this ratio, often without reaching statistical significance. Although both APD₉₀ shortening and ERP prolongation would individually be expected to increase PRR—and their combined effect would theoretically amplify this increase—the high within-group variability inherent to PRR as a derived difference measure (ERP–APD₉₀) likely limits statistical power to detect these changes. As reflected by the wide standard deviations at baseline (e.g., 13 ± 20 ms for lidocaine), variability in both APD₉₀ and ERP propagates into the PRR calculation, a limitation also observed in comparable Langendorff studies [26, 27]. Despite the lack of formal statistical significance in several groups, the numerically consistent trend toward PRR prolongation across all four drug groups suggests a class-wide effect attributable to sodium channel blockade by lidocaine and mepivacaine [28] and the additional potassium channel-blocking effects reported for bupivacaine and ropivacaine [21]. The observed differences in post-repolarization refractoriness among the investigated local anesthetics may in part reflect their distinct sodium channel recovery kinetics. Lidocaine and mepivacaine are characterized by rapid recovery from sodium channel block during diastole, allowing near-complete channel availability between action potentials at physiological heart rates. In contrast, bupivacaine exhibits slow recovery from sodium channel block during diastole—a so-called ‘fast-in, slow-out’ mechanism – resulting in cumulative block and persistent post-repolarization refractoriness, as reflected by a greater increase in the ERP/APD ratio in our data.

### Cardiotoxicity of Different Local Anesthetics

Perfusion with all four local anesthetics did not significantly increase ventricular tachycardia episodes, indicating no notable proarrhythmic potential for these agents. at the level of intrinsic cardiac function. However, in a whole-system context, proarrhythmic potential may be greater, as local anesthetics can also modulate autonomic nervous system activity [10]. At lower concentrations, transient sympathetic stimulation may increase heart rate and spatial dispersion of repolarization [12]—both established contributors to ventricular arrhythmogenesis—while at higher concentrations, impaired sympathetic reflexes and direct inhibition of β-adrenergic signaling [30]—particularly described for bupivacaine—may further destabilize cardiac electrophysiology beyond what is measurable in the denervated Langendorff model.

Nevertheless, the finding of low proarrhythmic risk in this model is crucial considering the increased dispersion and prolonged QT interval, both linked to higher susceptibility to ventricular arrhythmias [12, 13]. This observation is consistent with previous isolated heart studies, which demonstrated proarrhythmic effects of local anesthetics only at considerably higher concentrations—arrhythmias in isolated guinea pig hearts were reported at bupivacaine concentrations of ~ 10 µM [31], well above the 1 µM used in the present study—indicating that the proarrhythmic potential of local anesthetics in the isolated heart is highly concentration-dependent. Notably, bupivacaine showed a clear trend toward increased ventricular tachycardia episodes (11 of 13 hearts at 1 µM), suggesting that the arrhythmogenic threshold may be approached even at clinically relevant doses. A possible reason for the overall lack of a significant rise in ventricular arrhythmias might be the prolongation of the PRR, as previous research suggests that a prolonged PRR can protect the myocardium from premature excitation and reduce ventricular arrhythmia risk [26, 27].

Regarding the cardiodepressive effects, there was a significant rise in LOC episodes under bupivacaine perfusion. Additionally, 5 µM ropivacaine caused a significant increase, while 10 µM ropivacaine did not reach significance due to high variability in experiments. Perfusions with lidocaine and mepivacaine did not lead to a significant increase in LOC episodes.

This appears to involve several mechanisms: mainly, bupivacaine and ropivacaine cause prolonged inhibition or tonic blockade of sodium channels in rabbit heart tissues, unlike lidocaine [32]. This tonic sodium channel blockade could explain why loss of capture occurs without prior tachyarrhythmia, especially with bupivacaine. Additionally, bupivacaine and ropivacaine are more lipophilic than lidocaine and mepivacaine, which may promote accumulation in heart tissue and enhance tissue-specific cardiotoxicity from sodium channel blockade.

These results align with other studies and clinical reports indicating higher cardiodepressive risks for bupivacaine and, to a lesser extent, for ropivacaine [7, 31, 34, 35].

### Clinical Implications

This study showed that local anesthetics influence cardiac electrophysiology and that their individual effects and manifestations vary. While a tendency toward an increase in QT interval, dispersion, and PRR appear to be a class effects, cardiotoxicity varies and is particularly severe with bupivacaine due to episodes of loss of capture. It should be noted that the drug concentrations used in this study are within the peak plasma levels seen in clinical use of bupivacaine [36, 37], ropivacaine [38], lidocaine [39], and mepivacaine [40]. Importantly, as the Langendorff perfusate contains no plasma proteins, the concentrations applied reflect free (unbound) drug concentrations, which represent the pharmacologically active fraction. Given the high protein binding of bupivacaine (~ 95%) and ropivacaine (~ 94%), the lower doses used in this study (0.5 µM and 5 µM, respectively) already correspond to or exceed estimated free peak plasma concentrations in clinical practice, suggesting that even the lower doses tested here are of direct clinical relevance. Consistently, the lower doses produced significant electrophysiological effects in the present study—including significant QT prolongation and loss-of-capture episodes with ropivacaine, and significant APD shortening with bupivacaine—supporting the notion that clinically relevant concentrations are sufficient to alter cardiac electrophysiology at the intrinsic level. Whether comparable effects manifest in the whole system at lower doses may depend on the interplay between direct cardiac effects and autonomic modulation, as discussed above. The present data provide mechanistic support for lidocaine’s role in antiarrhythmic therapy: perfusion with lidocaine produced a significant shortening of APD₉₀—consistent with its known class Ib antiarrhythmic effect via late sodium channel inhibition [18]—without significant prolongation of the QT interval, indicating absence of proarrhythmic repolarization delay. Simultaneously, the effective refractory period was significantly prolonged, and a trend toward PRR prolongation was observed, both of which are associated with protection against premature excitation and reentrant arrhythmias. Importantly, lidocaine did not significantly increase ventricular tachycardia or loss-of-capture episodes, underscoring its favorable electrophysiological safety profile at clinically relevant concentrations. Taken together, these findings are consistent with lidocaine’s established role in drug-based antiarrhythmic therapy [2, 3]. Systemic exposure to bupivacaine, which most commonly occurs as a consequence of accidental intravascular injection during regional anesthesia rather than intentional systemic administration, carries a pronounced risk of severe cardiac toxicity as reflected by our findings. Therefore, extreme caution is warranted in clinical settings where inadvertent intravascular injection is possible. For example, it is advisable to use bupivacaine solely in anesthesia procedures with the lowest possible risk of accidental intravascular administration. The risk of local anesthetic toxicity should always be considered, and close patient monitoring along with appropriate treatment options should be prepared and implemented if necessary.

## Data Availability

The data supporting the findings of this study are available on request from the corresponding author.
